# MeCP2 Regulates the Synaptic Expression of a Dysbindin-BLOC-1 Network Component in Mouse Brain and Human Induced Pluripotent Stem Cell-Derived Neurons

**DOI:** 10.1371/journal.pone.0065069

**Published:** 2013-06-04

**Authors:** Jennifer Larimore, Pearl V. Ryder, Kun-Yong Kim, L. Alex Ambrose, Christopher Chapleau, Gaston Calfa, Christina Gross, Gary J. Bassell, Lucas Pozzo-Miller, Yoland Smith, Konrad Talbot, In-Hyun Park, Victor Faundez

**Affiliations:** 1 Department of Biology, Agnes Scott College, Decatur, Georgia, United States of America; 2 Cell Biology, Emory University, Atlanta, Georgia, United States of America; 3 Department of Genetics, Yale Stem Cell Center, Yale School of Medicine, New Haven, Connecticut, United States of America; 4 Department of Neurobiology, The University of Alabama, Birmingham, Alabama, United States of America; 5 Department of Neurology, Emory University, Atlanta, Georgia, United States of America; 6 Yerkes National Primate Center, Emory University, Atlanta, Georgia, United States of America; 7 Center for Neurobiology and Behavior, Department of Psychiatry, University of Pennsylvania, Philadelphia, Pennsylvania, United States of America; University of Insubria, Italy

## Abstract

Clinical, epidemiological, and genetic evidence suggest overlapping pathogenic mechanisms between autism spectrum disorder (ASD) and schizophrenia. We tested this hypothesis by asking if mutations in the ASD gene *MECP2* which cause Rett syndrome affect the expression of genes encoding the schizophrenia risk factor dysbindin, a subunit of the biogenesis of lysosome-related organelles complex-1 (BLOC-1), and associated interacting proteins. We measured mRNA and protein levels of key components of a dysbindin interaction network by, quantitative real time PCR and quantitative immunohistochemistry in hippocampal samples of wild-type and *Mecp2* mutant mice. In addition, we confirmed results by performing immunohistochemistry of normal human hippocampus and quantitative qRT-PCR of human inducible pluripotent stem cells (iPSCs)-derived human neurons from Rett syndrome patients. We defined the distribution of the BLOC-1 subunit pallidin in human and mouse hippocampus and contrasted this distribution with that of symptomatic *Mecp2* mutant mice. Neurons from mutant mice and Rett syndrome patients displayed selectively reduced levels of pallidin transcript. Pallidin immunoreactivity decreased in the hippocampus of symptomatic *Mecp2* mutant mice, a feature most prominent at asymmetric synapses as determined by immunoelectron microcopy. Pallidin immunoreactivity decreased concomitantly with reduced BDNF content in the hippocampus of *Mecp2* mice. Similarly, BDNF content was reduced in the hippocampus of BLOC-1 deficient mice suggesting that genetic defects in BLOC-1 are upstream of the BDNF phenotype in *Mecp2* deficient mice. Our results demonstrate that the ASD-related gene *Mecp2* regulates the expression of components belonging to the dysbindin interactome and these molecular differences may contribute to synaptic phenotypes that characterize *Mecp2* deficiencies and ASD.

## Introduction

Autism spectrum disorder (ASD) and schizophrenia are disorders with some intersecting clinical characteristics such as their shared impairment of social cognition [1]–[4]. Phenotypic similarities between these disorders suggest common molecular roots [5]. This hypothesis has recently received substantial support from epidemiological, bioinformatic, and genetic studies [6]. Epidemiological evidence points to non-genetic and genetic risk factors. Among the non-genetic factors, obstetric complications as well as migrant status increase the risk of both schizophrenia and autism [7] while among genetic risk factors a parental history of schizophrenia increases the risk for ASD and advanced paternal age is a risk factor for both schizophrenia and ASD [8]–[10]. Advanced paternal age can be explained by the increased rate of *de novo* mutations during spermatogenesis in older subjects [9]. Genome-wide association studies further support common molecular roots between schizophrenia and ASD. An increasing number of copy number variations that span multiple genes associate with both schizophrenia and ASD [11], [12]. This supportive genetic evidence extends to monogenic defects such as those in *GPHN* [13], ANK3 [14], *NRXN1* or *MECP2*. *NRXN1* encodes a presynaptic neuronal cell adhesion molecule [15], [16] and *NRXN1* genetic defects robustly associate with schizophrenia and ASD [17]–[21]. Similarly, mutations in the X-linked *MECP2* gene, which encodes the transcriptional regulator methyl-CpG-binding protein 2 (MeCP2), result in one of the ASDs, the Rett syndrome, and are associated with childhood schizophrenia [22]–[26]. Genetic manipulation of *Mecp2* in mice causes well-characterized synaptic phenotypes and a transcriptional signature, which is defined from mRNA expression profiles in loss- and gain-of-function mouse mutations [22], [27]. Mutations to *Mecp2* affect the neuronal expression of 12–15% of the mouse genome [28], [29], suggesting that some of these MeCP2-regulated transcripts could encode unrecognized synaptic proteins associated with schizophrenia pathogenesis. Here, we focus on a MeCP2-dependent mechanism that regulates the expression of subunits of the *b*iogenesis of *l*ysosome related *o*rganelles *c*omplex 1 (BLOC-1), a synaptic complex that contains the schizophrenia-associated protein, dysbindin.

Dysbindin and its protein interaction network regulate mechanisms implicated in schizophrenia pathogenesis pathways. Polymorphisms in *DTNBP1*, the gene encoding dysbindin, associate with schizophrenia [30]–[32]. Moreover, a significant reduction of synaptic dysbindin expression has been reported in nearly 80% of schizophrenia cases [33]–[36]. Dysbindin is part of a protein interaction network spanning a minimum of 24 experimentally identified proteins. Nearly one-third of the genes encoding these dysbindin network components are affected by copy number variation defects in schizophrenia subjects [37]. Functions ascribed to dysbindin further support its participation in schizophrenia disease mechanisms. Dysbindin is part of the octameric protein complex BLOC-1, which is involved in the targeting of membrane proteins from endosomes to diverse organelles and the synapse [31], [32], [38], [39]. Dysbindin localizes to pre- and post-synaptic compartments. At the pre-synaptic level, its deficiency affects synaptic homeostasis, synaptic vesicle composition and vesicle fusion, while at the post-synaptic level, its deficiency alters the surface content of glutamatergic and dopaminergic receptors, which are strongly implicated in schizophrenia pathogenesis [40]–[49]. The synaptic mechanisms in which the dysbindin network participates as well as the experimentally verified nature of this protein-protein interaction network make it a good target for exploring mechanistic convergence between ASD and schizophrenia. Here we test this idea by investigating if MeCP2 regulates the expression of components of the dysbindin protein interaction network. To this end, we first defined the normal anatomical and ultrastructural distribution of the BLOC-1 subunit pallidin in human and mouse hippocampus. Next, we demonstrate that *Mecp2* mice (*Mecp2^tm1.1.Jae/y^*) expressing a non-functional truncated protein possess reduced levels of pallidin transcript and synaptic protein expression in the hippocampal formation. Moreover, we recapitulated this reduced pallidin mRNA phenotype in human iPSCs differentiated into neurons from Rett syndrome patients carrying mutations in *MECP2*. The impaired pallidin expression phenotype in *Mecp2* mutant mice is most prominent at asymmetric synapses. We determined that *Mecp2* and BLOC-1 deficiencies share a reduced content of brain-derived neurotrophic factor (BDNF), suggesting that BLOC-1 is upstream of the BDNF phenotype in MeCP2-deficient brain. These findings reveal a novel molecular link between an ASD causative gene, *Mecp2*, and the protein interactome of the schizophrenia susceptibility gene product, dysbindin. We speculate that defects on the dysbindin interactome may contribute to synaptic and circuit defects that characterize *Mecp2* mutations.

## Materials and Methods

### Reagents

Mouse-anti pallidin was a gift from Dr. Esteban Dell’Angelica (UCLA, Los Angeles, California) [50] and rabbit anti-VAMP-2 was purchased from Synaptic Systems (Göttingen, Germany). Synaptophysin (SY38) antibody was from Chemicon International/Millipore (Billerica, MA, USA). Rabbit polyclonal antibodies against BDNF were from Santa Cruz (Dallas, Texas. SC-5456) and human recombinant BDNF was from Promega (Madison, WI. G1491). The monoclonal antibody specificity in brain immunohistochemistry was determined in this study using pallidin-null *Bloc1s6^pa/pa^* brain. B6.Cg*^pa^*/J breeding pairs were obtained from Jackson Labs (Bar Harbor, Maine). Breeding pairs of *Mecp2* mice [51] were purchased from the Mutant Mouse Regional Resource Center at the University of California, Davis (B6.Cg-*Mecp2* ^tm1.1Jae^, “Jaenisch” strain maintained in C57BL/6 background), and a colony established at UAB. Mice were bred in-house following Emory and UAB IUCAC approved protocols.

### Quantitative Real Time PCR (qRT-PCR)

Control and mutant cortical and hippocampal regions were dissected from P7 animals, which are asymptomatic, and young adult animals between P42–P52 sacrificed by CO2 narcosis. Tissue was then flash frozen. Following TRIzol (Invitrogen Life Technologies, Grand Island, NY) extraction, isolated mRNA was reverse transcribed into cDNA using SuperScript III First-Strand Synthesis (Invitrogen Life Technologies, Grand Island, NY). PCR amplifications were performed on a LightCycler480 Real Time plate reader using LightCycler 480 SYBR Green reagents (Roche, Indianapolis, IN). Table 1 describes the primers used in this study.

**Table 1:** qRT-PCR Primers Used in this Study and Animals Tested.

	*Forward*	*Reverse*	*Adult Brain* a	*P7 Brain* a	*IPSCs Lines*
***Bloc1s1***	AGCAGAACGAACGCAAGG	TTGAGGTGATCCACCAACG	5∶5:4∶3	3∶3:3∶3	
***Bloc1s2***	AATGGCCACATATTTGACTGG	TTCATATTTTCCAGGAGTTTGTAGTC	5∶5:5∶3	3∶3:3∶3	
***Bloc1s3***	GCCTAGGCTGTCACTCTGTGT	GGTGGTACGAATGTTCACCA	4∶4:3∶3	3∶3:3∶3	
***Muted***	GAGAACAGGAACGGAAAAAGG	TCCGCTCACGAACTCCTC	5∶5:5∶4	3∶3:3∶3	
***Pallidin***	CTCCAGACGGGGTCCTTAC	AGTCCTTCATCTGGAGACGTG	5∶5:5∶4	3∶3:3∶3	
***SNAPIN***	CCGGATCAATGAGGATCAGA	CCGCCTTAGTCGTTCCTGT	5∶5:5∶3	3∶3:3∶3	
***Dysbindin***	GATCGCAGAGAGGCGAGA	TGAGATGTCCATCAGGTCCA	4∶5:5∶5	3∶3:3∶3	
***BDNF***	AGTCTCCAGGACAGCAAAGC	TGCAACCGAAGTATGAAATAACC	4∶4:		
***PLDN***	AACTCACACAGAACCAAGTTGT	GGCATGATAGTGTTTAGCCTCAG			2
***BLOC1S1***	AGGAGGCGAGAGGCTATCAC	GGACCTGTAGGGTCTTCACCT			2
***TUBBS***	GCCGCTACCTGACGGTGGC	GGGCGGGATGTCACACACGG			2
***GFAP***	TGCTCGCCGCTCCTACGTCT	ATCCACCCGGGTCGGGAGTG			2
***NEUROD1***	AGGTGGTGCCTTGCTATTCT	TTGCAAAGCGTCTGAACGAA			2

a Numbers show IPSCs or animals analyzed in Figure 2, 3, 7 and correspond to Wild type hippocampus: *Mecp2* or BLOC-1 mutant hippocampus: Wild type cortex: *Mecp2* mutant cortex. Each animal qRT-PCR determination was performed at least in duplicate.

### Brain Sections, Immunohistochemistry and Microscopy

Detailed procedures for mouse tissue preparation, immunoperoxidase light microscopy, indirect immunofluorescence microscopy, immunoperoxidase electron microscopy and quantification procedures were described in our previous work [37], [43], [44].

Briefly, brain slice preparations were obtained from mice between 6 to 8 weeks of age. Following deep anesthesia with ketamine, animals were transcardially perfused with Ringer’s solution followed by fixative (4% paraformaldehyde with 0.1% glutaraldehyde). Following postfixation, their brains were cut into 60 µm thick sections and processed to localize pallidin at the light and electron microscopic level using mouse anti-Pallidin antibodies (1∶400), the peroxidase-anti-peroxidase (PAP) method and 3,3′ diaminobenzidine (DAB) as chromogen for the peroxidase reaction, according to procedures described in detail in our recent studies [37], [43], [44].

For light microscopy, sections were mounted on gelatin-coated slides, dehydrated, and coverslipped. Light microscopy analysis was performed with a Leica DMRB microscope (Leica Microsystems, Inc., Bannockburn, IL, USA) and images were captured with a CCD camera (Leica DC500). Images were acquired with a 10x/0.3 DiC objective. Images were acquired with Leica IM50 software.

Electron microscopy sections were further postfixed in osmium tetroxide, dehydrated and embedded in resin on microscope slides according to procedures described in our recent studies [37], [43], [44]. From this EM-prepared tissue, blocks of the dentate gyrus were mounted on resin blocks, faced with a glass knife, and sectioned with a diamond knife into 60 nm sections with an ultramicrotome (Leica Ultracut T2). Sections were collected on Pioloform-coated copper grids and stained with lead citrate for 5 minutes to enhance contrast on the electron microscope. Electron microscopy was performed with a Zeiss EM-10C and a JEOLL electron microscopes equipped with CCD cameras (DualView 300 W; Gatan, Inc., Pleasanton, CA, USA). Images were acquired with Gatan Digital Micrograph Software (v. 3.10.1; Gatan, Inc.) Ultrathin sections from the surface of the blocks of the dentate gyrus corresponding to regions where the neuropil and cell bodies intersect were chosen. Random fields of view including asymmetric synapses were imaged at 30 000–50 000X. A total of 25 fields were examined in each animal or a total of 70–100 fields was analyzed per genotype. The proportion of terminals with pallidin-immunoreactive active zones and spines with labeled PSDs was estimated in control and mutant mice.

The human hippocampus tissue was obtained from non-psychiatric individuals. Human hippocampus data is representative data from 19 normal human cases (9 males, 10 females) studied. The mean age +/− the SD was 81.11+/−9.8 years. The mean postmortem interval (PMI) +/− the SD was 11.34+/−9.2 hours. After removal of the hippocampal formation, the tissue was fixed for 12–24 hours in either neutral buffered formalin or ethanol in saline. Following embedding in paraffin, the tissue was sectioned at 10 µm, mounted on adhesive-coated slides, air dried, and then processed immunohistochemically for pallidin using a monoclonal antibody described above (1∶150), heat-induced epitope retrieval with EDTA, and a standard avidin-biotin peroxidase with light silver intensification following the protocol of Talbot et al. (2004) [33].

### Immunofluorescence Microscopy

Confocal microscopy was performed with an Axiovert 100 M microcope (Carl Zeiss) coupled to an Argon and HeNe1 lasers. Images were acquired using Plan Apochromat 10x/0.5 dry, 20×/0.5 dry, and 40×/1.3 and 63×/1.4 DiC oil objectives. Emission filters used for fluorescence imaging were BP 505–530 and LP 560. Images were acquired with ZEN and LSM 510 software (Carl Zeiss). Hippocampal formation 60 µm-thick brain sections were rinsed with PBS and then incubated in 1% sodium borohydride in PBS for 20 minutes at room temperature. Samples were pre-incubated in a solution of PBS with 5% normal horse serum and 1% BSA and 0.3% Triton X-100 for 60 minutes at room temperature. Samples were incubated overnight at 4°C in primary antibody solutions of PBS with 1% NHS and 1% BSA. After rinsing in PBS, sections were incubated for 60 minutes in a secondary antibody PBS solution with 1% NHS and 1% BSA and 1∶500 dilutions of the following Alexa-conjugated secondary antibodies: anti-mouse 555 and anti-rabbit 488 (Invitrogen Molecular Probes, Carlsbad, CA, USA). Following PBS rinses, sections were incubated in cupric sulfate (3.854 W/V Ammonium Acetate, 1.596 W/V Cupric Sulfate in distilled water, pH 5) for 30 minutes and mounted on slides with Vectashield (Vector Laboratories).

### Light Microscopy Quantitations

Quantitations of peroxidase light microscopy staining and immunofluorescence were performed as previously described using Metamorph software [43]–[45].

### Human iPSCs Cells and Neuronal Differentiation

The monoallelic expressing Rett syndrome iPSCs cells RTT3 and RTT4 were previously characterized [52]. iPSCs cells were differentiated into neural rosettes and neurons as described by Li and Zhang [53]. Neurons were generated from these cells by interference with bone morphogenetic protein signaling [54].

### Mouse and Human Subjects

Animal Procedures were approved by the IACUC committee at Emory University and the University of Alabama. Data on human postmortem tissue derived from samples of U.S. citizens autopsied at the Hospital of the University of Pennsylvania as approved by the Institutional Review Board at that university. Autopsy consent from next-of-kin or legal guardian was obtained in all cases. For most cases, consent was granted in writing before death and always confirmed after death. Ethics committee at the University of Pennsylvania approved consent procedures. To keep postmortem delays to a minimum when written consent had not been obtained before death, verbal consent was obtained as witnessed by a third party and documented by the physician making the request. Written records of the consent for autopsy were archived. These procedures for written and verbal consent are standard medical practice in the U.S.A.

### Statistical and Bioinformatic Analyses

Statistical analyses were performed with Synergy KaleidaGraph v4.03 (Reading, PA) or the Vassar Web Engine (http://vassarstats.net/). BLOC-1 interacting proteins were compiled from our quantitative dysbindin interactome and a previously published curated list of putative candidate interactors [37], [55]. A BLOC-1 network interaction map was built using GeneGo Metacore (version 6.11 build 41105) and visualized in Cytoscape (version 2.8.3). Node color and sizes were mapped to changes in mRNA expression level in *MECP2*-overexpressing and *Mecp2*–null mouse neuronal cells as previously reported [29].

## Results

### Bioinformatic Analysis Identify BLOC-1 Components among MeCP2-regulated Transcripts

**Figure 1:**
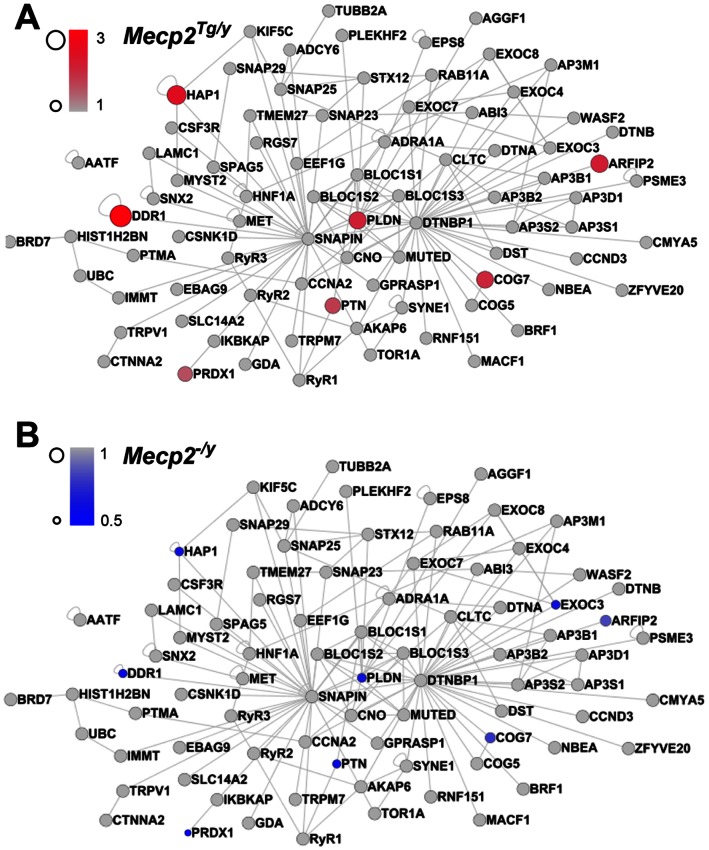
MeCP2 Regulates mRNA Levels of BLOC1-Interacting Proteins. BLOC1-interacting proteins were compiled from previous reports and mapped using the GeneGo Metacore pathway. The network was visualized in Cytoscope. Node sizes and colors were mapped to mRNA expression level changes in *MECP2*-overexpressing (A, *Mecp2^Tg/y^*) and *Mecp2* null mouse (B, *Mecp2^−/y^*) neurons as reported in Chahrour et al., 2008 [29]. We observed that six components of the BLOC1 interactome were modified by Mecp2 gene dosage. The protein products of three of these affected genes, PLDN, COG7, and PRDX1, are decreased in mice null for the schizophrenia susceptibility factor dysbindin [37].

The similarities between ASD and schizophrenia could result from a gene product affected in ASD. Such a gene could in turn modify the expression of proteins associated with pathways implicated in schizophrenia. We tested this hypothesis by investigating if the expression of genes encoding components of the dysbindin protein interaction network is susceptible to MeCP2 levels. We built a comprehensive dysbindin network by merging our dysbindin protein interaction network of 24 candidates with a prioritized list of putative protein-protein interactions for BLOC-1 subunits [37], [55]. The resultant dysbindin network contained 119 proteins. Six of these dysbindin interactome components were common to gene products whose mRNA expression is sensitive to Mecp2 content as previously determined by microarrays (Fig. 1) [29]. Pallidin (Pldn), peroxiredoxin 1 (Prdx1), COG7, pleiotrophin (PTN), ADP-ribosylation factor interacting protein 2 (ARFIP2), huntingtin-associated protein 1 (HAP1), and discoidin domain receptor tyrosine kinase 1 (DDR1) mRNAs are increased in *MECP2* over-expressing mouse neurons (Fig. 1A, *Mecp2^Tg/y^*) and decreased in *Mecp2*-null mouse neurons (Fig. 1B, *Mecp2^−/y^*). We previously demonstrated that three of these six proteins, pallidin, peroxiredoxin 1, and COG7, interact with dysbindin and their brain expression is sensitive to a dysbindin-null allele (*Bloc1s8^sdy/sdy^* previously referred as *Dtnbp1^sdy/sdy^*) [37]. Our bioinformatic analysis suggests that MeCP2 regulates expression of key components of the dysbindin interaction network.

**Figure 2:**
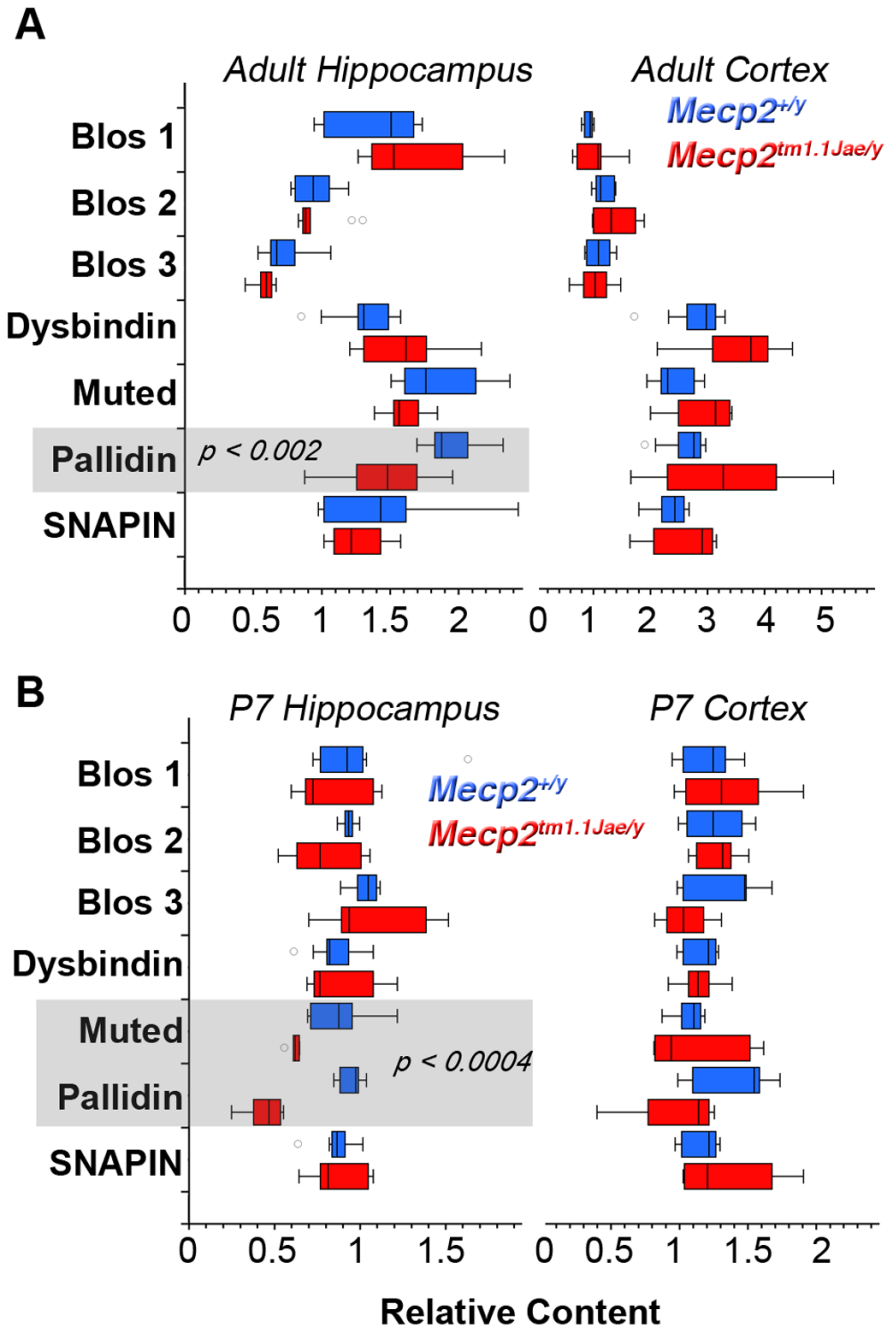
Quantitative Real Time PCR Determination of BLOC-1 Subunit Transcripts. BLOC-1 subunit transcripts from symptomatic adult (A) and P7 (B) mice hippocampi and cortices were analyzed by qRT-PCR. Box plot depicts relative mRNA content for wild type (*Mecp2^+/y^*, Blue) and *Mecp2* mutant tissue (*Mecp2^tm1.1Jae/y^*, Red). P values were obtained by Mann-Whitney U test. Number of animals tested is presented in Table 1.

We used quantitative real time PCR to determine the mRNA levels of transcripts encoding dysbindin and other BLOC-1 subunits in samples from the hippocampus and cortex from symptomatic young adult *Mecp2^tm1.1Jae/y^* mice and littermate controls. The mRNA levels of pallidin decreased by 26% in the hippocampus of symptomatic *Mecp2^tm1.1Jae/y^* mice as compared to controls (p<0.002 Mann-Whitney U test). In contrast, Bloc1s1–3, dysbindin, and muted transcripts were not affected in *Mecp2^tm1.1Jae/y^* hippocampus. The reduced content of pallidin was restricted to the hippocampus since all BLOC-1 subunit transcripts remained unaffected in *Mecp2^tm1.1Jae/y^* cortex (Fig. 2A). Since Rett syndrome affects the brain during development, we explored if BLOC-1 subunit mRNA levels were susceptible to *Mecp2* deficiency before becoming symptomatic [56], [57]. We examined mice at postnatal day seven, P7, a period that coincides with enhanced hippocampal synaptogenesis [58] and when alterations in spine formation occur in the *Mecp2* mutant mice [59]. The BLOC-1 pallidin subunit transcript was 50.5% lower in *Mecp2^tm1.1Jae/y^* P7 hippocampi, a statistically significant decrease. Mouse mutants carrying one copy of a null allele of pallidin (*Bloc1s6^+/pa^*) showed a 50% reduction of pallidin mRNA (data not shown), indicating that the effect of Mecp2-mutant upon pallidin mRNA expression is equivalent to the loss of one copy of the gene encoding pallidin. Strikingly, muted mRNA was also diminished in Mecp2 mutant hippocampi (Fig. 2B, 70% of control, p<0.0004 Mann-Whitney U test). P7 cortical transcripts encoding BLOC-1 subunits were not affected by the *Mecp2* mutant allele (Fig. 2B). These data demonstrate that MeCP2 regulates the expression of transcripts encoding BLOC-1 subunits in an anatomic and temporal-specific manner.

### Pallidin Transcript Content Decreases in Human *MECP2*-deficient Neurons

**Figure 3:**
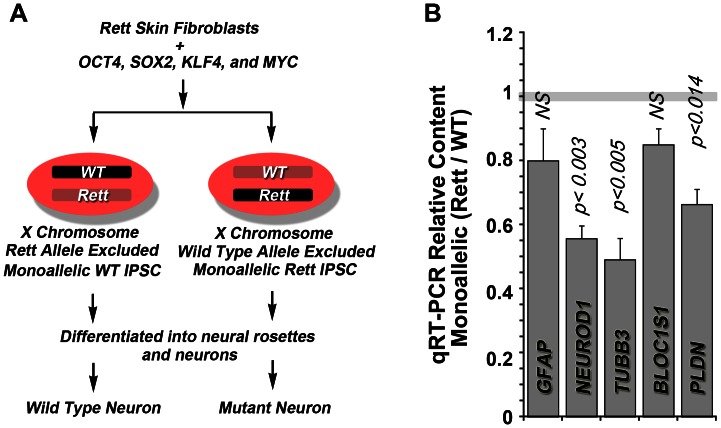
Quantitative Real Time PCR Determination of Pallidin Transcripts in Neurons derived from Human IPSCs. (A) Experimental design to generate monoallellic neurons from *MECP2* mutant Rett syndrome patient cells. (B) Wild type and mutant monoallellic IPSCs from the same patient were differentiated into neurons and analyzed by qRT-PCR. Plot depicts ratio of mRNA content between the wild type and mutant monoallellic cells from the same subject (n = 2 patients). P values were obtained by One Way Anova with Dunnett’s Multiple comparisons correction.

We sought to determine if the pallidin mRNA phenotypes observed in *Mecp2^tm1.1Jae/y^* mouse hippocampus were observed in Rett syndrome human neural tissue. We took advantage of neuronal cells differentiated from human iPSCs cells generated from skin fibroblasts of Rett syndrome patients. Fibroblasts were reprogramed into iPSCs by infecting them with a retrovirus expressing OCT4, SOX2, KLF4, and MYC. *MECP2* is encoded in the X-chromosome, the status of which is maintained after reprogramming (Fig. 3A) [60]. This chromosomal inactivation allowed us to generate IPSC clones from the same patient where either the wild-type or the MeCP2 mutant alleles were selectively expressed [52]. This approach facilitates transcript expression analysis since control and experimental samples are from the same individual, thus eliminating contributions by inter-individual genome differences [52].

We focused on patients carrying single *MECP2* alleles, E235fs or the R306C, and generated monoallellic expressing iPSC lines that we differentiated into neurons. We validated these cells by recapitulating a neuronal maturation defect we have previously reported [52]. This phenotype is characterized by a 50% decrease in the neuronal specific markers NeuroD1 and the neuron specific beta tubulin III, TuJ, encoded by *TUBB3* (Fig. 3B). Pallidin mRNA was significantly reduced by 35% in neurons monoallellically expressing mutant MeCP2 (Fig. 3B). However, the mRNA levels of the BLOC-1 subunit, Bloc1s1, were not significantly different compared to neurons expressing the wild type allele of *MECP2* or a control transcript (GFAP). These results demonstrate that defects in the expression of pallidin mRNA observed in *Mecp2^tm1.1Jae/y^* mice are common to those of patients carrying two different mutant alleles of *MECP2*.

### Anatomical Distribution of Mecp2 in Mouse and Human Brain

**Figure 4:**
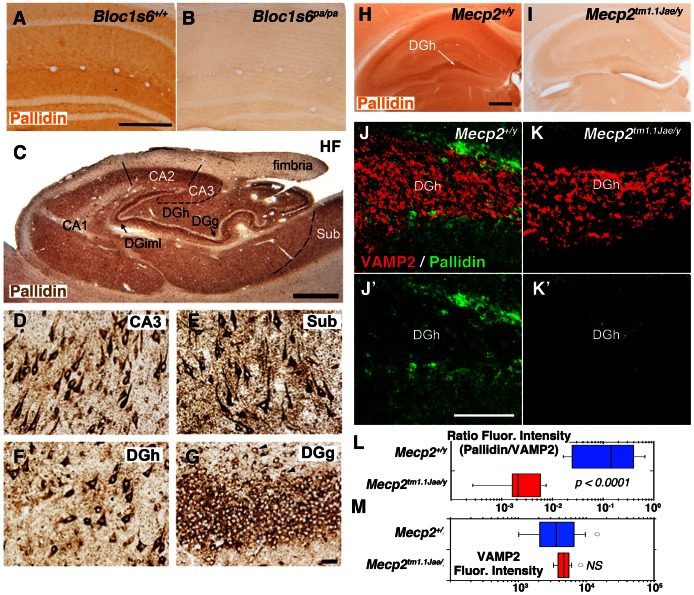
Light Immunomicroscopy of Pallidin in Mouse Hippocampus. *Images A, B, H-K’ depict sections from mouse hippocampus*. Sections correspond to immunoperoxidase microscopy with a pallidin monoclonal antibody in wild type (*Bloc1s6^+/+^*, A or *Mecp2^+/y^*, H), pallidin null (*Bloc1s6^pa/pa^*, B) and *Mecp2* mutant hippocampi (*Mecp2^tm1.1Jae/y^*, I). J–K’ depict indirect immunofluorescence microscopy of Pallidin and VAMP2. Quantitative imaging was performed by confocal microscopy of wild type (*Mecp2^+/y^*, J–J′) and *Mecp2* mutant hippocampus (*Mecp2^tm1.1Jae/y^*, K, K′). VAMP2 was used as a control to normalize staining between animals and experiments. L–M) Box plots depict relative fluorescence intensity expressed as a ratio between pallidin and VAMP2 and the total VAMP2 fluorescence intensity in the dentate gyrus, respectively. Wild type (*Mecp2^+/y^*, Blue, n = 4) and *Mecp2* mutant tissue (*Mecp2^tm1.1Jae/y^*, Red, n = 4) were analyzed. P values were obtained by Mann-Whitney U test. The VAMP2 content is similar between genotypes. Scale bars A = 0.5 mm, H = 1 mm, J′ = 25 µm. DGh, dentate gyrus hilus. *Images C–G correspond to sections from human hippocampal tissue stained with pallidin antibody*. In C, HF denotes the hippocampal formation, consisting of the hippocampus proper (CA1–3), the dentate gyrus (DG), and the subiculum (Sub). The inner molecular layer of the DG (C, DGiml), the DG hilus (DGh) and the dentat gyrus granule cells layer (DGg) are indicated. Pallidin immunoreactivity is present in cell bodies of CA3 cells (D), subicular pyramidal cells (E), dentate gyrus hilus (F), and dentate gyrus granule cells (G). The presence of pallidin in ectopic granule cells (arrow heads in G) verifies that granule cells, as opposed to terminal fields among them, contain the protein. Note that pallidin is a cytoplasmic, not a nuclear protein. The scale bar in C is 1 mm; that in G is 50 µm.

In order to assess the consequences of a *Mecp2* mutation upon pallidin hippocampal protein expression, we first determined the anatomical localization of pallidin, which has not been reported in the mammalian brain. To this end, we used a monoclonal antibody against pallidin in adult mouse and human hippocampal tissue (Fig. 4) [61]. We established the specificity of this antibody in tissue sections from wild-type and pallidin-null *Bloc1s6^pa/pa^* brain, as demonstrated by the loss of immuno-peroxidase labeling in pallidin-null brain (Fig. 4, compare A–B). We then examined the distribution of pallidin in human hippocampal tissue. Strong pallidin immunoreactivity was observed throughout the neuropil of the human hippocampal formation (Fig. 4C, HF). Similar to the distribution of dysbindin we previously reported [33], the highest neuropil levels of pallidin were found in axon terminal fields of glutamatergic neurons intrinsic to the hippocampal formation, most conspicuously in the inner molecular layer of the dentate gyrus (Fig. 4C, DGiml) and among CA2 and CA3 pyramidal cells (Fig. 4C). Figures 4D–G also show that neurons producing these axon terminal fields (hippocampal pyramidal cells in CA3 and polymorph cells in the dentate gyrus hilus [DGh]) are also rich in pallidin (Fig. 4 D, F), as are pyramidal cells in the subiculum (Fig. 4E). Pallidin in dentate gyrus granule cells are clearly seen in Fig. 4G. The distribution of pallidin in mouse brain was similar to the human hippocampus, with a prominent staining in the neuropil of the dentate gyrus hilus and synaptic fields such as the molecular layer of the dentate gyrus (Fig. 4A and H).

**Figure 5:**
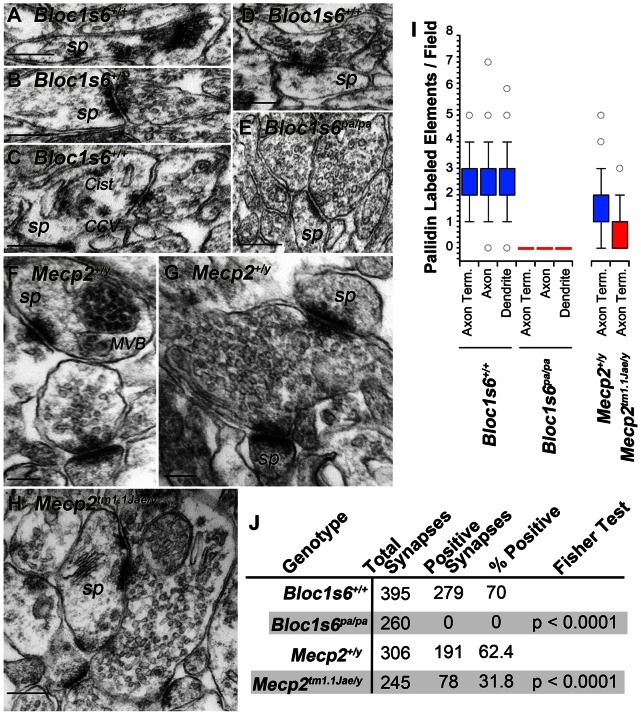
Electron Immunomicroscopy of Pallidin in Mouse Dentate Gyrus. Images depict immunoperoxidase electron microscopy with a pallidin monoclonal antibody in wild type (*Bloc1s6^+/+^*, A–D or *Mecp2^+/y^*, F–G), pallidin null (*Bloc1s6^pa/pa^*, E) and *Mecp2* mutant hippocampi (*Mecp2^tm1.1Jae/y^*, H). A–H depict representative asymmetric axospinous synapses from the hilus of the dentate gyrus. *Sp*, spine; *MBV*, multivesicular body; *CCV* clathrin coated vesicle; *Cist*, cisterna. Structures were identified following defined ultrastructural criteria [88]. I) Box plot depicts quantitation of pallidin immuno-positive synaptic compartments in wild type (*Bloc1s6^+/+^ Mecp2^+/y^*, Blue, n = 3), pallidin null (*Bloc1s6^pa/pa^*, Red, n = 1) and *Mecp2* mutant tissue (*Mecp2^tm1.1Jae/y^*, Red, n = 3). Dendrite includes immuno-positive spines as well as dendritic shafts. J) Synapse count and numbers of pallidin immuno-positive synapses per genotype. Bars 200 nm.

Dysbindin is localized to presynaptic and postsynaptic compartments yet if other subunits of the BLOC-1 complex are localized to the synapse remains unknown [40]. Immunofluorescence microscopy revealed the presence of pallidin concentrated in cell bodies of dentate gyrus neurons and as a diffuse staining in the adjacent neuropil (Fig. 4J–J′). A similar pattern was observed by immuno-peroxidase staining (Fig. 4F). The low neuropil pallidin signal attained precluded colocalization studies with a universal presynaptic marker, the synaptic vesicle protein VAMP2 (Fig. 4J–J′) [62]. Thus to address the subcellular localization of pallidin, we performed quantitative immuno-electron peroxidase microscopy of the dentate gyrus and determined the ultrastructural distribution of pallidin in wild-type and pallidin-null *Bloc1s6^pa/pa^* hippocampal tissue. Pallidin immunoreactivity was present in axon terminals, axons, and dendritic spines in the dentate gyrus of wild-type animals (Figs. 5A–D, F–G, I–J). Pallidin immunoreactivity decorated synaptic vesicles closely apposed to the active zone (Fig. 5A–D), coated vesicles or cisternae present in dendritic spines (CCV and Cist, respectively, Fig. 5C), and internal vesicles of multivesicular bodies located in spines (MVB, Fig. 5F). None of these structures were labeled in pallidin-null *Bloc1s6^pa/pa^* tissue (Fig. 5, I–J). These results indicate that pallidin is present in pre- and post-synaptic compartments consistent with a role of BLOC-1 and its protein interaction network modulating synaptic physiology.

This anatomical and ultrastructural distribution of pallidin in human and mouse hippocampus was contrasted between wild type and *Mecp2* mutant mouse tissue. Notably, we observed a marked reduction of pallidin immunoreactivity in the dentate gyrus, CA1 and CA3 as well as their associated synaptic fields in *Mecp2^tm1.1Jae/y^* hippocampus (Fig. 4, compare H–I). We performed quantitative confocal microscopy of the dentate gyrus labeled with antibodies directed against pallidin and a control marker not affected by BLOC-1 or *Mecp2* mutant alleles, the synaptic vesicle protein VAMP2 (VAMP2, Fig. 4 compare J–K) [43], [62]–[64]. Similar to the immuno-peroxidase results, pallidin content was reduced in the dentate gyrus of *Mecp2^tm1.1Jae/y^* mice (Fig. 4, L) without affecting the VAMP2 content (Fig. 4, M). We next asked if the reduction of pallidin content affected the synapse. We quantified the number of asymmetric synapses in wild type and symptomatic adult *Mecp2^tm1.1Jae/y^* mice that were positive for pallidin immunoreactivity by immunoelectron microscopy. Total synaptic counts per unit area showed no significant difference between control and *Mecp2* or *Bloc1s6* null genotypes (Fig. 5J). However, symptomatic *Mecp2^tm1.1Jae/y^* mice demonstrated a 50% reduction in the number of pallidin-positive presynaptic elements in asymmetric synapses of the dentate gyrus (Fig. 5F–G compare to H, and I–J). Our results show that pallidin protein expression phenotypes exceed those predicted by the content of pallidin transcripts in adult *Mecp2^tm1.1Jae/y^* hippocampus and demonstrate that MeCP2 regulates the synaptic expression of a BLOC-1 network component.

### Effects of Mecp2 and BLOC-1 Genetic Deficiencies on BLOC-1 Cargo Content in the Dentate Gyrus

**Figure 6:**
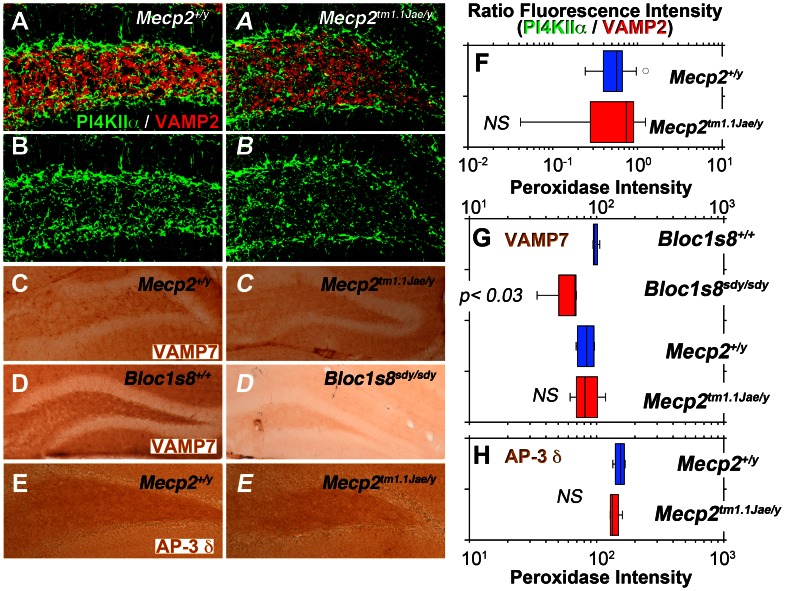
*Mecp2* Deficiency Does Not Affect the Content of BLOC-1 Sensitive Markers in the Dentate Gyrus. Images depict indirect quantitative immunofluorescence microscopy of PI4KIIα and VAMP2 (A–B) or immunoperoxidase light microscopy of VAMP7 (C–D) or the AP-3 δ subunit (E). A–E italic letters represent mutant *Mecp2* and BLOC-1 *Bloc1s8^sdy/sdy^*. F–H Box plots depict the quantitation of immunoreactivities of antigens presented in A–E and *A–E*. P values were obtained by Mann-Whitney U test, n = 4 independent stainings from 2 animals per genotype.

Reduced pallidin immunoreactivity in *Mecp2^tm1.1Jae/y^* dentate gyrus suggests impaired BLOC-1-dependent trafficking in *Mecp2^tm1.1Jae/y^* hippocampus. We and others have established that BLOC-1 and its binding partner, the adaptor complex AP-3, generate vesicles that target the SNARE VAMP7 and phosphatidylinositol-4-kinase type IIα (PI4KIIα) to nerve terminals [43]–[45], [65]. Mutations in BLOC-1 subunits lead to reduced AP-3, PI4KIIα, and VAMP7 immunoreactivity in axons and/or asymmetric axospinous nerve terminals in the dentate gyrus [43], [44], [45]. Consistently, AP-3, VAMP7, and PI4KIIα are found in synaptic vesicles [64], [66], [67]. Thus, we tested if *Mecp2* mutations would lead to reduced AP-3, PI4KIIα, and/or VAMP7 immunoreactivity in the dentate gyrus secondary to decreased pallidin content. The immunoreactivity of PI4KIIα (Fig. 6A to 6*B*), VAMP7 (Fig. 6C and *C*), and the delta subunit of AP-3 (AP-3δ, Fig. 6E and *E*), were not significantly modified in *Mecp2^tm1.1Jae/y^* dentate gyrus. In contrast, VAMP7 immunoreactivity in dysbindin-null mice (*Bloc1s8^sdy/sdy^*, Fig. 6D–*D*) was robustly decreased in *Bloc1s8^sdy/sdy^*, much like the phenotype previously observed in BLOC-1 null mice *Bloc1s6^pa/pa^* or *Bloc1s5^mu/mu^* [44]. These results suggest that genetic defects in *Mecp2* do not affect the delivery of BLOC-1 cargoes to synaptic vesicles.

**Figure 7:**
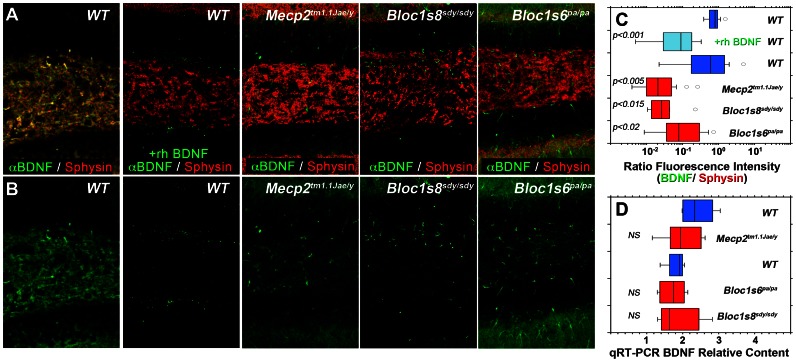
*Mecp2* and BLOC-1 Deficiency Affect BDNF Immunoreactivity in the Dentate Gyrus. Images depict indirect quantitative immunofluorescence confocal microscopy of BDNF and the synaptic vesicle marker synaptophysin in the dentate gyrus from *Mecp2^tm1.1Jae/y^*, the BLOC-1-null mice *Bloc1s8^sdy/sdy^*, and *Bloc1s6^pa/pa^* (A–B). BDNF immunorreactivity was abrogated by preincubation of antibodies with recombinant human BDNF (rhBDNF, A–B). Box plots in C depict the quantitation of BDNF immunoreactivity normalized to synaptophysin. P values were obtained by One Way Anova with Dunnett’s Multiple comparisons correction, n = 4 independent stainings from 2 animals per genotype. D, BDNF transcripts from symptomatic adult mice hippocampi were analyzed by qRT-PCR. Box plot depicts relative mRNA content for wild type (*Mecp2^+/y^*, Blue), *Mecp2* mutant tissue (*Mecp2^tm1.1Jae/y^*, Red), as well as the BLOC-1-null mice *Bloc1s8^sdy/sdy^*, and *Bloc1s6^pa/pa^*. P values were obtained by One Way Anova with Dunnett’s Multiple comparisons correction, n = 4 animals per genotype.

Defective BLOC-1 function in *Bloc1s8^sdy/sdy^* mice results in alterations in the structure and fusion of large dense secretory granules [46]. Thus, we tested the hypothesis that neuropeptides implicated in the pathogenesis of ASD and/or schizophrenia could be commonly affected in *Mecp2* and BLOC-1 genetic defects. We focused on BDNF, a neurotrophic factor packaged in large dense core vesicles present in pre- and post-synaptic terminals whose content is reduced in diverse brain regions of *Mecp2*-deficient mice [68]–[71]. We measured the immunoreactivity of BDNF and the synaptic vesicle protein synaptophysin by quantitative confocal immunofluorescence microscopy in dentate gyruses from *Mecp2^tm1.1Jae/y^*, the BLOC-1-null mice *Bloc1s8^sdy/sdy^*, and *Bloc1s6^pa/pa^* (Fig. 7). BDNF antibody signal localized in the pre-synaptic terminal as indicated by overlap with the synaptic vesicle marker synaptophysin (Fig. 7A, Sphysin). BDNF immunoreactivity specificity was confirmed by out-competition of the dentate gyrus BDNF immunoreactivity with recombinant human BDNF (Fig. 7A–B and in C compare blue and light blue boxes, rhBDNF). Similar to previous reports, we also observed a pronounced reduction of BDNF immunoreactivity in *Mecp2^tm1.1Jae/y^* dentate gyrus (Fig. 7A–B and in C compare blue to red boxes) [68], [71]. Importantly and analogous to *Mecp2^tm1.1Jae/y^* dentate gyrus, the content of BDNF was reduced in the dentate of animals carrying null alleles of the BLOC-1 complex, *Bloc1s8^sdy/sdy^* and *Bloc1s6^pa/pa^* (Fig. 7A–B and in C compare blue to red boxes). These changes in BDNF immunoreactivity in *Mecp2* and BLOC-1 deficient dentate gyrus were not due to changes in BDNF mRNA content as determined by qRT-PCR (Fig. 7D compare blue to red boxes). These results demonstrate that genetic defects in Mecp2 and BLOC-1 protein function generate a common BDNF phenotype, a growth factor packaged into large secretory granules present in nerve terminals.

## Discussion

Phenotypic overlap between ASD and schizophrenia is likely rooted in shared molecular pathways between both disorders [5]. This concept is supported by recent epidemiological evidence suggesting that parental history of schizophrenia is a risk factor for ASD and advanced paternal age is a risk factor for both schizophrenia and ASD [8]–[10], [72]. Moreover, genetic data reaffirm common molecular roots to both disorders either at the copy number variations level [11] or the disease phenotypes observed in monogenic defects [17]–[21], [23], [26]. Here we expand the mechanisms that could explain similarities between these disorders testing the idea that molecular phenotypes downstream of an ASD monogenic defect would encompass synaptic gene products associated with schizophrenia. Our results reveal a novel regulatory pathway linking MeCP2, an epigenetic transcriptional regulator and an ASD-Rett syndrome causative gene, with the interactome of the schizophrenia susceptibility gene product dysbindin. We demonstrate that a mutation in Mecp2 in mice and humans alters pallidin hippocampus transcript levels and in mouse, protein content at the synapse. The mRNA phenotype in mice is more pronounced in asymptomatic postnatal day 7 animals, a time associated with synapse formation in hippocampal ontogenesis [58]. These findings suggest that altered function of pallidin and/or the dysbindin pathway could contribute to part of the synaptic defects that characterize MeCP2 deficiencies in asymptomatic and symptomatic stages of disease [27], [63], [73]–[75]. This hypothesis is consistent with the reported functions of BLOC-1 complex subunits in neuronal cells. Pallidin- or dysbindin-deficient neurons or neuroblastoma cells have impaired neuritogenesis *in vitro* [76]–[78]. In addition, dysbindin-null neurons and chromaffin cells have defective fusion of synaptic vesicles and large dense core granules, respectively [46]. These cellular defects likely contribute to impaired paired-pulse facilitation in dysbindin-null neurons [79] and the defective synaptic scaling observed in dysbindin- or snapin-deficient *Drosophila* synapses [41], [42]. Strikingly, MeCP2 regulates synaptic scaling, suggesting that BLOC-1 subunits may underlie part of the scaling phenotypes in Mecp2 mutant neurons [80].

Dysbindin localizes to pre- and post-synaptic compartments in mammalian neurons, yet before our study there were no reports of the subcellular localization of pallidin in mammalian brain [40]. Pallidin immunoreactivity is present in synaptic fields in human and mouse hippocampus. This light microscopy observation is confirmed by immunoelectron microscopy where we detect pallidin on synaptic vesicles in terminals forming asymmetric synapses of the dentate gyrus as well as on cisternae and multivesicular bodies in dendritic spines. This ultrastructural subcellular distribution confirms biochemical studies of BLOC-1 subunits co-purifying with synaptic vesicles and synaptosomes [34], [44], [81]. Moreover, pallidin localization to MVBs supports the role of BLOC-1 complexes controlling surface expression and lysosomal delivery of post-synaptic receptors [48], [82]. *Mecp2* mutation decreases the mRNA levels of pallidin by 26% in the hippocampus of symptomatic animals and by ∼30% in iPSCs-derived neurons from patients carrying *MECP2* pathogenic mutations. Pallidin protein immunoreactivity by light and electron immunomicroscopy is even more reduced in the neuropil and cell bodies of neurons in the dentate gyrus. This enhanced pallidin reduction suggests additional factors downstream of MeCP2 control the protein expression of pallidin. The identity of such mechanisms remains unknown. Immunoelectron microscopy indicates that 70% of asymmetric axospinous synapses in the *Mecp2* mutant mice have no detectable pallidin in the dentate gyrus. Other synaptic fields in the hippocampus such as the stratum lucidum in CA3 also have decreased pallidin immunoreactivity, suggesting that the synaptic defect observed in the dentate gyrus might be common to other synapses. The number of synapses per unit area of the dentate gyrus is not affected in *Mecp2*-deficient mice as assessed by either ultrastructural synapse counting or staining with the synaptic vesicle marker VAMP2 (Fig. 3–4). This result is similar to previous electron microscopy findings in CA1 [57]. Thus, we interpret this decreased content of pallidin-positive asymmetric axospinous synapses as decreased expression of pallidin in synapses rather than decreased synapse density. Importantly, loss of just one copy of dysbindin is sufficient to trigger synaptic and behavioral phenotypes in mice [49], [79], supporting the contention that a 50% reduction in the levels of pallidin observed by immunoelectron microscopy in *Mecp2* mutant hippocampus may contribute to synaptic and circuit defects that characterize *Mecp2* mutations [27], [63], [73]–[75].

The reduced pallidin immunoreactivity in *Mecp2*-deficient hippocampus suggested that BLOC-1 sensitive cargoes delivered to nerve terminals would be affected in the dentate gyrus of *Mecp2^tm1.1Jae/y^* mice. We tested this hypothesis by analyzing the expression of three proteins present in regulated secretory vesicles: VAMP7 and PI4KIIα, which reside in synaptic vesicles and whose targeting is dependent on BLOC-1 and AP-3 complexes, and BDNF, which is present in large dense core granules [83]–[85]. We focused on BDNF because its brain content is reduced in *Mecp2* brains [68]–[71]. However, if brain BDNF is affected by BLOC-1 deficiency was unknown. Of these three secretory vesicle markers, only BDNF immunoreactivity was reduced to the absence of BLOC-1 in the dentate gyrus. In the past we have interpreted reduced levels of an antigen in BLOC-1-null dentate gyrus as an indication of defective membrane traffic [43]. However, our findings with BDNF need additional evidence to determine if the reduced BDNF immunoreactivity in the dentate gyrus is a reflection of a BLOC-1-defective trafficking mechanism.

Mutations of dysbindin are associated with a destabilization of other components of the BLOC-1 interactome [37], [86], [87]. For example, dysbindin-null hippocampi possess reduced levels AP-3, peroxiredoxin 1, and COG7 [37], [43], [44]. It is possible that MeCP2 deficiency may also influence other components of the BLOC-1 interactome as indicated in Fig. 1. In fact, the levels of muted are reduced in *Mecp2* deficient P7 hippocampi. We postulate that diverse components of the BLOC-1 interactome may be targets for transcriptional regulation by MeCP2 and/or may be directly affected in *de novo* cases of ASD or other neurodevelopmental disorders.

## References

[1] Korkmaz B (2011) Theory of mind and neurodevelopmental disorders of childhood. Pediatr Res 69: 101R–108R. 21289541 10.1203/PDR.0b013e318212c177

[2] Sugranyes G, Kyriakopoulos M, Corrigall R, Taylor E, Frangou S (2011) Autism spectrum disorders and schizophrenia: meta-analysis of the neural correlates of social cognition. PLoS One 6: e25322. 21998649 10.1371/journal.pone.0025322 PMC3187762

[3] King BH, Lord C (2011) Is schizophrenia on the autism spectrum? Brain Res 1380: 34–41. 21078305 10.1016/j.brainres.2010.11.031

[4] Lugnegard T, Unenge Hallerback M, Hjarthag F, Gillberg C (2013) Social cognition impairments in Asperger syndrome and schizophrenia. Schizophr Res 143: 277–284. 23266067 10.1016/j.schres.2012.12.001

[5] Moreno-De-Luca A, Myers SM, Challman TD, Moreno-De-Luca D, Evans DW, et al (2013) Developmental brain dysfunction: revival and expansion of old concepts based on new genetic evidence. Lancet Neurol 12: 406–414. 23518333 10.1016/S1474-4422(13)70011-5 PMC4013791

[6] Cristino AS, Williams SM, Hawi Z, An JY, Bellgrove MA, et al.. (2013) Neurodevelopmental and neuropsychiatric disorders represent an interconnected molecular system. Mol Psychiatry. 10.1038/mp.2013.16 23439483

[7] Hamlyn J, Duhig M, McGrath J, Scott J (2012) Modifiable risk factors for schizophrenia and autism - Shared risk factors impacting on brain development. Neurobiol Dis. 10.1016/j.nbd.2012.10.023 23123588

[8] Sullivan PF, Magnusson C, Reichenberg A, Boman M, Dalman C, et al.. (2012) Family History of Schizophrenia and Bipolar Disorder as Risk Factors for AutismFamily History of Psychosis as Risk Factor for ASD. Arch Gen Psychiatry: 1–5. 10.1001/archgenpsychiatry.2012.730 PMC4187103 22752149

[9] Kong A, Frigge ML, Masson G, Besenbacher S, Sulem P, et al (2012) Rate of de novo mutations and the importance of father’s age to disease risk. Nature 488: 471–475. 22914163 10.1038/nature11396 PMC3548427

[10] Buizer-Voskamp JE, Laan W, Staal WG, Hennekam EA, Aukes MF, et al (2011) Paternal age and psychiatric disorders: findings from a Dutch population registry. Schizophr Res 129: 128–132. 21489755 10.1016/j.schres.2011.03.021 PMC3110532

[11] Doherty JL, O’Donovan MC, Owen MJ (2012) Recent genomic advances in schizophrenia. Clin Genet 81: 103–109. 21895634 10.1111/j.1399-0004.2011.01773.x

[12] Consortium C-DGotPG (2013) Identification of risk loci with shared effects on five major psychiatric disorders: a genome-wide analysis. Lancet. 10.1016/S0140-6736(12)62129-1 PMC3714010 23453885

[13] Lionel AC, Vaags AK, Sato D, Gazzellone MJ, Mitchell EB, et al.. (2013) Rare exonic deletions implicate the synaptic organizer Gephyrin (GPHN) in risk for autism, schizophrenia and seizures. Hum Mol Genet. 10.1093/hmg/ddt056 23393157

[14] Iqbal Z, Vandeweyer G, van der Voet M, Waryah AM, Zahoor MY, et al.. (2013) Homozygous and heterozygous disruptions of ANK3: at the crossroads of neurodevelopmental and psychiatric disorders. Hum Mol Genet. 10.1093/hmg/ddt043 23390136

[15] Rapoport JL, Giedd JN, Gogtay N (2012) Neurodevelopmental model of schizophrenia: update 2012. Mol Psychiatry 17: 1228–1238. 22488257 10.1038/mp.2012.23 PMC3504171

[16] Zoghbi HY, Bear MF (2012) Synaptic dysfunction in neurodevelopmental disorders associated with autism and intellectual disabilities. Cold Spring Harb Perspect Biol 4. 10.1101/cshperspect.a009886 PMC3282414 22258914

[17] Kirov G, Gumus D, Chen W, Norton N, Georgieva L, et al (2008) Comparative genome hybridization suggests a role for NRXN1 and APBA2 in schizophrenia. Hum Mol Genet 17: 458–465. 17989066 10.1093/hmg/ddm323

[18] Kirov G, Rujescu D, Ingason A, Collier DA, O’Donovan MC, et al (2009) Neurexin 1 (NRXN1) deletions in schizophrenia. Schizophr Bull 35: 851–854. 19675094 10.1093/schbul/sbp079 PMC2728827

[19] Ching MS, Shen Y, Tan WH, Jeste SS, Morrow EM, et al (2010) Deletions of NRXN1 (neurexin-1) predispose to a wide spectrum of developmental disorders. Am J Med Genet B Neuropsychiatr Genet 153B: 937–947. 20468056 10.1002/ajmg.b.31063 PMC3001124

[20] Schaaf CP, Boone PM, Sampath S, Williams C, Bader PI, et al.. (2012) Phenotypic spectrum and genotype-phenotype correlations of NRXN1 exon deletions. Eur J Hum Genet. 10.1038/ejhg.2012.95 PMC3499754 22617343

[21] Gauthier J, Siddiqui TJ, Huashan P, Yokomaku D, Hamdan FF, et al (2011) Truncating mutations in NRXN2 and NRXN1 in autism spectrum disorders and schizophrenia. Hum Genet 130: 563–573. 21424692 10.1007/s00439-011-0975-z PMC3204930

[22] Chahrour M, Zoghbi HY (2007) The story of Rett syndrome: from clinic to neurobiology. Neuron 56: 422–437. 17988628 10.1016/j.neuron.2007.10.001

[23] Lam CW, Yeung WL, Ko CH, Poon PM, Tong SF, et al (2000) Spectrum of mutations in the MECP2 gene in patients with infantile autism and Rett syndrome. J Med Genet 37: E41. 11106359 10.1136/jmg.37.12.e41 PMC1734495

[24] Amir RE, Van den Veyver IB, Wan M, Tran CQ, Francke U, et al (1999) Rett syndrome is caused by mutations in X-linked MECP2, encoding methyl-CpG-binding protein 2. Nat Genet 23: 185–188. 10508514 10.1038/13810

[25] Calfa G, Percy AK, Pozzo-Miller L (2011) Experimental models of Rett syndrome based on Mecp2 dysfunction. Exp Biol Med (Maywood) 236: 3–19. 21239731 10.1258/ebm.2010.010261 PMC3059199

[26] Cohen D, Lazar G, Couvert P, Desportes V, Lippe D, et al (2002) MECP2 mutation in a boy with language disorder and schizophrenia. Am J Psychiatry 159: 148–149. 10.1176/appi.ajp.159.1.148-a 11772708

[27] Na ES, Nelson ED, Kavalali ET, Monteggia LM (2012) The Impact of MeCP2 Loss- or Gain-of-Function on Synaptic Plasticity. Neuropsychopharmacology. 10.1038/npp.2012.116 PMC3521965 22781840

[28] Tudor M, Akbarian S, Chen RZ, Jaenisch R (2002) Transcriptional profiling of a mouse model for Rett syndrome reveals subtle transcriptional changes in the brain. Proc Natl Acad Sci U S A 99: 15536–15541. 12432090 10.1073/pnas.242566899 PMC137752

[29] Chahrour M, Jung SY, Shaw C, Zhou X, Wong ST, et al (2008) MeCP2, a key contributor to neurological disease, activates and represses transcription. Science 320: 1224–1229. 18511691 10.1126/science.1153252 PMC2443785

[30] Ayalew M, Le-Niculescu H, Levey DF, Jain N, Changala B, et al.. (2012) Convergent functional genomics of schizophrenia: from comprehensive understanding to genetic risk prediction. Mol Psychiatry. 10.1038/mp.2012.37 PMC3427857 22584867

[31] Ghiani CA, Dell’angelica EC (2011) Dysbindin-containing complexes and their proposed functions in brain: from zero to (too) many in a decade. ASN Neuro 3: e00058. 21504412 10.1042/AN20110010 PMC3155195

[32] Talbot K, Ong WY, Blake DJ, Tang D, Louneva N, et al.. (2009) Dysbindin-1 and its protein family, with special attention to the potential role of dysbindin-1 in neuronal functions and the pathophysiology of schizophrenia. In: Kantrowitz Ja, editor. Handbook of Neurochemistry and Molecular Neurobiology. New York: Springer Science. 107–241.

[33] Talbot K, Eidem WL, Tinsley CL, Benson MA, Thompson EW, et al (2004) Dysbindin-1 is reduced in intrinsic, glutamatergic terminals of the hippocampal formation in schizophrenia. J Clin Invest 113: 1353–1363. 15124027 10.1172/JCI20425 PMC398430

[34] Talbot K, Louneva N, Cohen JW, Kazi H, Blake DJ, et al (2011) Synaptic dysbindin-1 reductions in schizophrenia occur in an isoform-specific manner indicating their subsynaptic location. PLoS ONE 6: e16886. 21390302 10.1371/journal.pone.0016886 PMC3046962

[35] Tang J, LeGros RP, Louneva N, Yeh L, Cohen JW, et al (2009) Dysbindin-1 in dorsolateral prefrontal cortex of schizophrenia cases is reduced in an isoform-specific manner unrelated to dysbindin-1 mRNA expression. Hum Mol Genet 18: 3851–3863. 19617633 10.1093/hmg/ddp329 PMC2748893

[36] Weickert CS, Rothmond DA, Hyde TM, Kleinman JE, Straub RE (2008) Reduced DTNBP1 (dysbindin-1) mRNA in the hippocampal formation of schizophrenia patients. Schizophr Res 98: 105–110. 17961984 10.1016/j.schres.2007.05.041 PMC2246024

[37] Gokhale A, Larimore J, Werner E, So L, Moreno-De-Luca A, et al (2012) Quantitative proteomic and genetic analyses of the schizophrenia susceptibility factor dysbindin identify novel roles of the biogenesis of lysosome-related organelles complex 1. J Neurosci 32: 3697–3711. 22423091 10.1523/JNEUROSCI.5640-11.2012 PMC3313842

[38] Ryder PV, Faundez V (2009) Schizophrenia: the “BLOC” may be in the endosomes. Sci Signal 2: pe66. 19843956 10.1126/scisignal.293pe66 PMC5696790

[39] Mullin AP, Gokhale A, Larimore J, Faundez V (2011) Cell biology of the BLOC-1 complex subunit dysbindin, a schizophrenia susceptibility gene. Mol Neurobiol 44: 53–64. 21520000 10.1007/s12035-011-8183-3 PMC3321231

[40] Talbot K, Cho DS, Ong WY, Benson MA, Han LY, et al (2006) Dysbindin-1 is a synaptic and microtubular protein that binds brain snapin. Hum Mol Genet 15: 3041–3054. 16980328 10.1093/hmg/ddl246

[41] Dickman DK, Davis GW (2009) The schizophrenia susceptibility gene dysbindin controls synaptic homeostasis. Science 326: 1127–1130. 19965435 10.1126/science.1179685 PMC3063306

[42] Dickman DK, Tong A, Davis GW (2012) Snapin is critical for presynaptic homeostatic plasticity. J Neurosci 32: 8716–8724. 22723711 10.1523/JNEUROSCI.5465-11.2012 PMC3395587

[43] Larimore J, Tornieri K, Ryder PV, Gokhale A, Zlatic SA, et al (2011) The schizophrenia susceptibility factor dysbindin and its associated complex sort cargoes from cell bodies to the synapse. Mol Biol Cell 22: 4854–4867. 21998198 10.1091/mbc.E11-07-0592 PMC3237628

[44] Newell-Litwa K, Chintala S, Jenkins S, Pare JF, McGaha L, et al (2010) Hermansky-Pudlak protein complexes, AP-3 and BLOC-1, differentially regulate presynaptic composition in the striatum and hippocampus. J Neurosci 30: 820–831. 20089890 10.1523/JNEUROSCI.3400-09.2010 PMC2824551

[45] Newell-Litwa K, Salazar G, Smith Y, Faundez V (2009) Roles of BLOC-1 and adaptor protein-3 complexes in cargo sorting to synaptic vesicles. Mol Biol Cell 20: 1441–1453. 19144828 10.1091/mbc.E08-05-0456 PMC2649275

[46] Chen XW, Feng YQ, Hao CJ, Guo XL, He X, et al (2008) DTNBP1, a schizophrenia susceptibility gene, affects kinetics of transmitter release. J Cell Biol 181: 791–801. 18504299 10.1083/jcb.200711021 PMC2396815

[47] Iizuka Y, Sei Y, Weinberger DR, Straub RE (2007) Evidence that the BLOC-1 protein dysbindin modulates dopamine D2 receptor internalization and signaling but not D1 internalization. J Neurosci 27: 12390–12395. 17989303 10.1523/JNEUROSCI.1689-07.2007 PMC6673263

[48] Tang TT, Yang F, Chen BS, Lu Y, Ji Y, et al (2009) Dysbindin regulates hippocampal LTP by controlling NMDA receptor surface expression. Proc Natl Acad Sci U S A 106: 21395–21400. 19955431 10.1073/pnas.0910499106 PMC2795512

[49] Karlsgodt KH, Robleto K, Trantham-Davidson H, Jairl C, Cannon TD, et al (2011) Reduced dysbindin expression mediates N-methyl-D-aspartate receptor hypofunction and impaired working memory performance. Biol Psychiatry 69: 28–34. 21035792 10.1016/j.biopsych.2010.09.012 PMC4204919

[50] Starcevic M, Dell’Angelica EC (2004) Identification of snapin and three novel proteins (BLOS1, BLOS2, and BLOS3/reduced pigmentation) as subunits of biogenesis of lysosome-related organelles complex-1 (BLOC-1). J Biol Chem 279: 28393–28401. 15102850 10.1074/jbc.M402513200

[51] Chen RZ, Akbarian S, Tudor M, Jaenisch R (2001) Deficiency of methyl-CpG binding protein-2 in CNS neurons results in a Rett-like phenotype in mice. Nat Genet 27: 327–331. 11242118 10.1038/85906

[52] Kim KY, Hysolli E, Park IH (2011) Neuronal maturation defect in induced pluripotent stem cells from patients with Rett syndrome. Proc Natl Acad Sci U S A 108: 14169–14174. 21807996 10.1073/pnas.1018979108 PMC3161557

[53] Li XJ, Zhang SC (2006) In vitro differentiation of neural precursors from human embryonic stem cells. Methods Mol Biol 331: 169–177. 16881517 10.1385/1-59745-046-4:168

[54] Gerrard L, Rodgers L, Cui W (2005) Differentiation of human embryonic stem cells to neural lineages in adherent culture by blocking bone morphogenetic protein signaling. Stem Cells 23: 1234–1241. 16002783 10.1634/stemcells.2005-0110

[55] Rodriguez-Fernandez IA, Dell’Angelica EC (2009) A data-mining approach to rank candidate protein-binding partners-The case of biogenesis of lysosome-related organelles complex-1 (BLOC-1). J Inherit Metab Dis 32: 190–203. 19083121 10.1007/s10545-008-1014-7 PMC2756288

[56] Guy J, Hendrich B, Holmes M, Martin JE, Bird A (2001) A mouse Mecp2-null mutation causes neurological symptoms that mimic Rett syndrome. Nat Genet 27: 322–326. 11242117 10.1038/85899

[57] Calfa G, Hablitz JJ, Pozzo-Miller L (2011) Network hyperexcitability in hippocampal slices from Mecp2 mutant mice revealed by voltage-sensitive dye imaging. J Neurophysiol 105: 1768–1784. 21307327 10.1152/jn.00800.2010 PMC3075283

[58] Mody M, Cao Y, Cui Z, Tay KY, Shyong A, et al (2001) Genome-wide gene expression profiles of the developing mouse hippocampus. Proc Natl Acad Sci U S A 98: 8862–8867. 11438693 10.1073/pnas.141244998 PMC37526

[59] Chapleau CA, Boggio EM, Calfa G, Percy AK, Giustetto M, et al (2012) Hippocampal CA1 Pyramidal Neurons of Mecp2 Mutant Mice Show a Dendritic Spine Phenotype Only in the Presymptomatic Stage. Neural Plast 2012: 976164. 22919518 10.1155/2012/976164 PMC3418521

[60] Tchieu J, Kuoy E, Chin MH, Trinh H, Patterson M, et al (2010) Female human iPSCs retain an inactive X chromosome. Cell Stem Cell 7: 329–342. 20727844 10.1016/j.stem.2010.06.024 PMC2935700

[61] Nazarian R, Starcevic M, Spencer MJ, Dell’Angelica EC (2006) Reinvestigation of the dysbindin subunit of BLOC-1 (biogenesis of lysosome-related organelles complex-1) as a dystrobrevin-binding protein. Biochem J 395: 587–598. 16448387 10.1042/BJ20051965 PMC1462696

[62] Baumert M, Maycox PR, Navone F, De Camilli P, Jahn R (1989) Synaptobrevin: an integral membrane protein of 18,000 daltons present in small synaptic vesicles of rat brain. EMBO J 8: 379–384. 2498078 10.1002/j.1460-2075.1989.tb03388.x PMC400817

[63] Asaka Y, Jugloff DG, Zhang L, Eubanks JH, Fitzsimonds RM (2006) Hippocampal synaptic plasticity is impaired in the Mecp2-null mouse model of Rett syndrome. Neurobiol Dis 21: 217–227. 16087343 10.1016/j.nbd.2005.07.005

[64] Takamori S, Holt M, Stenius K, Lemke EA, Gronborg M, et al (2006) Molecular anatomy of a trafficking organelle. Cell 127: 831–846. 17110340 10.1016/j.cell.2006.10.030

[65] Scheuber A, Rudge R, Danglot L, Raposo G, Binz T, et al (2006) Loss of AP-3 function affects spontaneous and evoked release at hippocampal mossy fiber synapses. Proc Natl Acad Sci U S A 103: 16562–16567. 17056716 10.1073/pnas.0603511103 PMC1637621

[66] Salazar G, Craige B, Wainer BH, Guo J, De Camilli P, et al (2005) Phosphatidylinositol-4-kinase type II alpha is a component of adaptor protein-3-derived vesicles. Mol Biol Cell 16: 3692–3704. 15944223 10.1091/mbc.E05-01-0020 PMC1182308

[67] Guo J, Wenk MR, Pellegrini L, Onofri F, Benfenati F, et al (2003) Phosphatidylinositol 4-kinase type IIalpha is responsible for the phosphatidylinositol 4-kinase activity associated with synaptic vesicles. Proc Natl Acad Sci U S A 100: 3995–4000. 12646710 10.1073/pnas.0230488100 PMC153036

[68] Li W, Calfa G, Larimore J, Pozzo-Miller L (2012) Activity-dependent BDNF release and TRPC signaling is impaired in hippocampal neurons of Mecp2 mutant mice. Proc Natl Acad Sci U S A 109: 17087–17092. 23027959 10.1073/pnas.1205271109 PMC3479462

[69] Kline DD, Ogier M, Kunze DL, Katz DM (2010) Exogenous brain-derived neurotrophic factor rescues synaptic dysfunction in Mecp2-null mice. J Neurosci 30: 5303–5310. 20392952 10.1523/JNEUROSCI.5503-09.2010 PMC3367888

[70] Wang H, Chan SA, Ogier M, Hellard D, Wang Q, et al (2006) Dysregulation of brain-derived neurotrophic factor expression and neurosecretory function in Mecp2 null mice. J Neurosci 26: 10911–10915. 17050729 10.1523/JNEUROSCI.1810-06.2006 PMC6674736

[71] Chang Q, Khare G, Dani V, Nelson S, Jaenisch R (2006) The disease progression of Mecp2 mutant mice is affected by the level of BDNF expression. Neuron 49: 341–348. 16446138 10.1016/j.neuron.2005.12.027

[72] Jokiranta E, Brown AS, Heinimaa M, Cheslack-Postava K, Suominen A, et al.. (2013) Parental psychiatric disorders and autism spectrum disorders. Psychiatry Res. 10.1016/j.psychres.2013.01.005 PMC3654001 23391634

[73] Chao HT, Zoghbi HY, Rosenmund C (2007) MeCP2 controls excitatory synaptic strength by regulating glutamatergic synapse number. Neuron 56: 58–65. 17920015 10.1016/j.neuron.2007.08.018 PMC2198899

[74] Chao HT, Chen H, Samaco RC, Xue M, Chahrour M, et al (2010) Dysfunction in GABA signalling mediates autism-like stereotypies and Rett syndrome phenotypes. Nature 468: 263–269. 21068835 10.1038/nature09582 PMC3057962

[75] Dani VS, Chang Q, Maffei A, Turrigiano GG, Jaenisch R, et al (2005) Reduced cortical activity due to a shift in the balance between excitation and inhibition in a mouse model of Rett syndrome. Proc Natl Acad Sci U S A 102: 12560–12565. 16116096 10.1073/pnas.0506071102 PMC1194957

[76] Ghiani CA, Starcevic M, Rodriguez-Fernandez IA, Nazarian R, Cheli VT, et al (2009) The dysbindin-containing complex (BLOC-1) in brain: developmental regulation, interaction with SNARE proteins and role in neurite outgrowth. Mol Psychiatry 15: 204–215. 10.1038/mp.2009.58 PMC2811213 19546860

[77] Ma X, Fei E, Fu C, Ren H, Wang G (2011) Dysbindin-1, a schizophrenia-related protein, facilitates neurite outgrowth by promoting the transcriptional activity of p53. Mol Psychiatry 16: 1105–1116. 21502952 10.1038/mp.2011.43

[78] Kubota K, Kumamoto N, Matsuzaki S, Hashimoto R, Hattori T, et al (2009) Dysbindin engages in c-Jun N-terminal kinase activity and cytoskeletal organization. Biochem Biophys Res Commun 379: 191–195. 19094965 10.1016/j.bbrc.2008.12.017

[79] Jentsch JD, Trantham-Davidson H, Jairl C, Tinsley M, Cannon TD, et al (2009) Dysbindin modulates prefrontal cortical glutamatergic circuits and working memory function in mice. Neuropsychopharmacology 34: 2601–2608. 19641486 10.1038/npp.2009.90 PMC2762021

[80] Qiu Z, Sylwestrak EL, Lieberman DN, Zhang Y, Liu XY, et al (2012) The Rett syndrome protein MeCP2 regulates synaptic scaling. J Neurosci 32: 989–994. 22262897 10.1523/JNEUROSCI.0175-11.2012 PMC3711796

[81] Salazar G, Craige B, Styers ML, Newell-Litwa KA, Doucette MM, et al (2006) BLOC-1 complex deficiency alters the targeting of adaptor protein complex-3 cargoes. Mol Biol Cell 17: 4014–4026. 16760431 10.1091/mbc.E06-02-0103 PMC1556383

[82] Marley A, von Zastrow M (2010) Dysbindin promotes the post-endocytic sorting of G protein-coupled receptors to lysosomes. PLoS ONE 5: e9325. 20174469 10.1371/journal.pone.0009325 PMC2824829

[83] Dieni S, Matsumoto T, Dekkers M, Rauskolb S, Ionescu MS, et al (2012) BDNF and its pro-peptide are stored in presynaptic dense core vesicles in brain neurons. J Cell Biol 196: 775–788. 22412021 10.1083/jcb.201201038 PMC3308691

[84] Matsuda N, Lu H, Fukata Y, Noritake J, Gao H, et al (2009) Differential activity-dependent secretion of brain-derived neurotrophic factor from axon and dendrite. J Neurosci 29: 14185–14198. 19906967 10.1523/JNEUROSCI.1863-09.2009 PMC3849773

[85] Dean C, Liu H, Staudt T, Stahlberg MA, Vingill S, et al (2012) Distinct subsets of Syt-IV/BDNF vesicles are sorted to axons versus dendrites and recruited to synapses by activity. J Neurosci 32: 5398–5413. 22514304 10.1523/JNEUROSCI.4515-11.2012 PMC3352930

[86] Li W, Zhang Q, Oiso N, Novak EK, Gautam R, et al (2003) Hermansky-Pudlak syndrome type 7 (HPS-7) results from mutant dysbindin, a member of the biogenesis of lysosome-related organelles complex 1 (BLOC-1). Nat Genet 35: 84–89. 12923531 10.1038/ng1229 PMC2860733

[87] Di Pietro SM, Dell’Angelica EC (2005) The cell biology of Hermansky-Pudlak syndrome: recent advances. Traffic 6: 525–533. 15941404 10.1111/j.1600-0854.2005.00299.x

[88] Peters A, Palay SL, Webster H (1991) Fine Structure of the Nervous System: Neurons and Their Supporting Cells: Oxford University Press, USA. 528 p.

